# Segmented Macular Layer Volumes from Spectral Domain Optical Coherence Tomography in 184 Adult Twins: Associations With Age and Heritability

**DOI:** 10.1167/iovs.61.5.44

**Published:** 2020-05-23

**Authors:** Ali Lamin, Zakariya Jarrar, Katie M. Williams, Anurag Garg, Khadijah Basheer, Sobha Sivaprasad, Christopher J. Hammond, Omar A. Mahroo

**Affiliations:** ^1^ Institute of Ophthalmology, University College London, London, United Kingdom; ^2^ Retinal Service, Moorfields Eye Hospital, London, United Kingdom; ^3^ Section of Academic Ophthalmology, King's College London, St Thomas' Hospital Campus, London, United Kingdom; ^4^ Department of Twin Research and Genetic Epidemiology, King's College London, London, United Kingdom

**Keywords:** retina, macula, photoreceptors, optical coherence tomography, twin study

## Abstract

**Purpose:**

To investigate segmented macular layer volumes from a healthy adult twin cohort (TwinsUK), exploring changes with age and heritability.

**Methods:**

Macular spectral domain optical coherence tomography images were acquired from monozygotic (MZ) and dizygotic (DZ) twins in a cross-sectional study. The following layer volumes were derived for circles of 3 and 6 mm diameter around the foveal center, using automated segmentation software: retinal nerve fiber layer (RNFL), ganglion cell–inner plexiform layer (GCIPL), inner nuclear layer (INL), outer plexiform layer (OPL), outer nuclear layer (ONL), photoreceptors (PR), retinal pigment epithelium (RPE), and total retinal volume (TRV). Correlation coefficients (intereye; age; intrapair for MZ and DZ pairs) were quantified; heritability was estimated using structural equation modeling.

**Results:**

Scans from 184 participants were included. Intereye correlation was highest for TRV and GCIPL. Negative correlations with age (for 3- or 6-mm areas, or both) were observed for TRV, RNFL, GCIPL, and INL. Positive correlations were observed for PR, RPE, and OPL. For all layers, intrapair correlation was greater for MZ than DZ pairs. Heritability estimates were highest (>80%) for TRV and GCIPL volume, and lowest for RPE volume.

**Conclusions:**

Although TRV was negatively correlated with age, all layers did not show negative correlation. Some inner layers thinned with age, whereas some outer volumes increased (not the ONL). Reduced RPE phagocytic function with age and remodeling in the OPL could be contributing factors. Heritability estimates were highest for inner retinal layers (particularly GCIPL), and lowest for RPE volume.

Spectral domain optical coherence tomography (OCT) yields a cross-sectional representation of the retinal layers allowing precise assessment of retinal structural pathology. Qualitative assessment of OCT images guides clinical diagnoses. For macular OCT, the quantitative measure traditionally used, in both clinical and research settings, is total retinal thickness, often divided into circular subfields around the foveal center. More recently, segmentation algorithms have allowed quantification of the thickness or volume of each layer separately, from the thickness of the retinal nerve fiber layer (RNFL) down to the thickness of the retinal pigment epithelium (RPE). This is of clinical and scientific interest as layers are often selectively attenuated in different retinal diseases. In addition, thinning of retinal layers can be associated with neurologic diseases^1^^,^^2^; thinning of the RNFL has been shown to be associated with cognitive impairment, with evidence that such measurements could have predictive value.^3^

Twin studies allow investigation of relative genetic and environmental contributions to phenotypic traits. By making measurements in monozygotic (MZ) and dizygotic (DZ) twin pairs, intrapair correlation can be compared: a significantly higher correlation in MZ twins indicates that genetic factors are important. Formal calculation of heritability by twin modeling permits estimation of the proportion of the variance in a trait that is attributable to genetic factors. Previous twin studies have demonstrated significant heritability for macular thickness,^4^ macular pigment optical density^5^^,^^6^ and spatial patterns,^6^^,^^7^ retinal vascular patterns^8^ and peripapillary RNFL.^9^ In the present study, we analyzed segmented layer volumes from macular OCT scans in a twin cohort using an automated segmentation algorithm to investigate heritability of each layer separately. We also explored associations with age and right-left eye correlations in the same cohort.

## Methods

### Participants

Participants were recruited from the TwinsUK registry based at St. Thomas’ Hospital. This is a cohort of largely healthy adult twins, who have volunteered for research studies.^10^ The participants in the present study were taking part in a larger electroretinography study.^11^

### Retinal Imaging and Segmentation

Macular OCT images were acquired from both eyes using a 6- x 6-mm macular cube scan (3D OCT; Topcon Corporation, Tokyo, Japan). Macular layer volumes were derived for circles of 3 and 6 mm diameter around the foveal center, using automated layer segmentation software (Orion; Voxeleron LLC, San Francisco, California, USA). The following layer volumes were derived: RNFL, ganglion cell–inner plexiform layer (GCIPL), inner nuclear layer (INL), outer plexiform layer (OPL), outer nuclear layer (ONL), photoreceptors (PR), RPE, and total retinal volume (TRV).

### Calculating Correlations

Coefficients of intrapair correlation were calculated for MZ and DZ twins. Pearson coefficients were used, with Spearman coefficients also calculated for any parameters found to differ significantly from a normal distribution (Kolmogorov-Smirnov test). Correlations with age were also calculated, as well as coefficients of intereye correlation for each parameter.

### Calculating Heritability

Age-adjusted heritability was estimated formally for each of the layer volumes (averaged between eyes for each participant), using maximum likelihood structural equation twin modeling as described previously,^12^ using the OpenMx package (https://openmx.ssri.psu.edu/) in the R statistical computing environment (http://www.r-project.org). The variance of a trait is estimated by some combination of the contributions from three factors: the additive genetic component (A); the shared environment (C) or the nonadditive genetic component (D); and the unique environment (E). Univariant ACE or ADE models were executed with standardized path coefficients and expected variance and covariance matrices. Goodness of fit of the full and reduced ACE and ADE models were compared with the observed data. The most parsimonious model to explain the observed variance was selected using the Akaike information criterion; this was identified as the AE model for most of the phenotypes. Heritability was calculated as the proportion of total variance of the trait (V) resulting from the additive genetic effect (A) in the best-fitting model.

### Ethical Approval

Participants gave informed consent. The study had local research ethics committee approval and was conducted in accordance with the tenets of the Declaration of Helsinki.

## Results

Macular OCT images from 184 participants (54 MZ pairs; 38 DZ pairs) were included for analysis. In four participants, the image from one eye only was used owing to a poor quality scan in the fellow eye. Mean (SD) age was 62.0 (11.1) years. For MZ pairs, mean (SD) age was 60.1 (11.6) years, and ranged from 32 to 84 years. For DZ pairs, mean (SD) age was 64.8 (10.0) years, and ranged from 36 to 86 years. MZ pairs were slightly younger (*P* = 0.044), and so age-adjusted heritability estimates were generated. The majority of twins were women (all of the DZ pairs, and 93% of the MZ pairs), reflecting the demographics of the TwinsUK cohort.

The majority of participants (>90%) were not known to have a retinal disorder. Fourteen participants (7.6%) had a history of one of the following conditions that could affect retinal layer volumes: age-related macular degeneration (1.6%), glaucoma (1.6%), glaucoma suspect/ocular hypertension (1.6%), previous retinal detachment (1.1%), vitreomacular traction (0.5%), and unspecified retinal problems (1.1%). In addition, three participants had unilateral amblyopia (1.6%), and five had diabetes (2.7%), but no detectable diabetic maculopathy.

### Mean Values and Correlations with Age

Table 1 shows means and standard deviations for the various layer volumes for the whole cohort. None of the parameters were found to differ significantly from a normal distribution, with the exception of RPE volume. Correlations with age are also given in Table 1. Here parameters from both twins were averaged for each pair, so that each pair contributed only once. Significant negative correlations with age were observed for TRV (for 3- and 6-mm circles), RNFL (6-mm circle), GCIPL (3 and 6 mm circles), and INL (3 mm).

**Table 1.** Segmented Layer Volumes, Correlations with Age, and Correlations Between Eyes

			Correlation with Age		
Circle		Mean (SD)	Correlation		Intereye Correlation
Diameter (mm)	Parameter	Value (mm^3^)	Coefficient	*P* Value	Coefficient
3	TRV	2.178 (0.098)	–0.270*	0.009	0.768*
	RNFL	0.209 (0.019)	–0.077	0.464	0.565*
	GCIPL	0.548 (0.053)	–0.499*	4.21 x 10^−7^	0.748*
	INL	0.289 (0.026)	–0.353*	5.52 x 10^−4^	0.633*
	OPL	0.195 (0.029)	0.194	0.064	0.417*
	ONL	0.625 (0.048)	–0.126	0.231	0.634*
	PR	0.314 (0.032)	0.364*	3.56 x 10^−4^	0.433*
	RPE	0.041 (0.070)	0.245* (0.231*)	0.018 (0.025)	0.469* (0.681*)
6	TRV	8.331 (0.371)	–0.335*	0.001	0.863*
	RNFL	1.158 (0.127)	–0.535*	4.01 x 10^−8^	0.754*
	GCIPL	1.967 (0.168)	–0.483*	1.07 x 10^−6^	0.835*
	INL	1.001 (0.084)	–0.157	0.134	0.748*
	OPL	0.688 (0.077)	0.245*	0.018	0.428*
	ONL	2.252 (0.130)	–0.163	0.119	0.774*
	PR	1.266 (0.099)	0.424*	2.50 x 10^−5^	0.508*
	RPE	0.144 (0.176)	0.260* (0.263*)	0.012 (0.011)	0.537* (0.673*)

Mean (SD) values are given for the whole cohort (*n* = 184). Correlations with age are given (with parameters from both twins averaged within each twin pair). Intereye correlations are given for the cohort. All intereye correlations were highly significant (*P* < 1 × 10^−8^). All are Pearson correlation coefficients, but for RPE parameters, Spearman coefficients are also given in parentheses as these parameters deviated from a normal distribution. *Denotes significance (*P* < 0.05).

**Figure 1.**
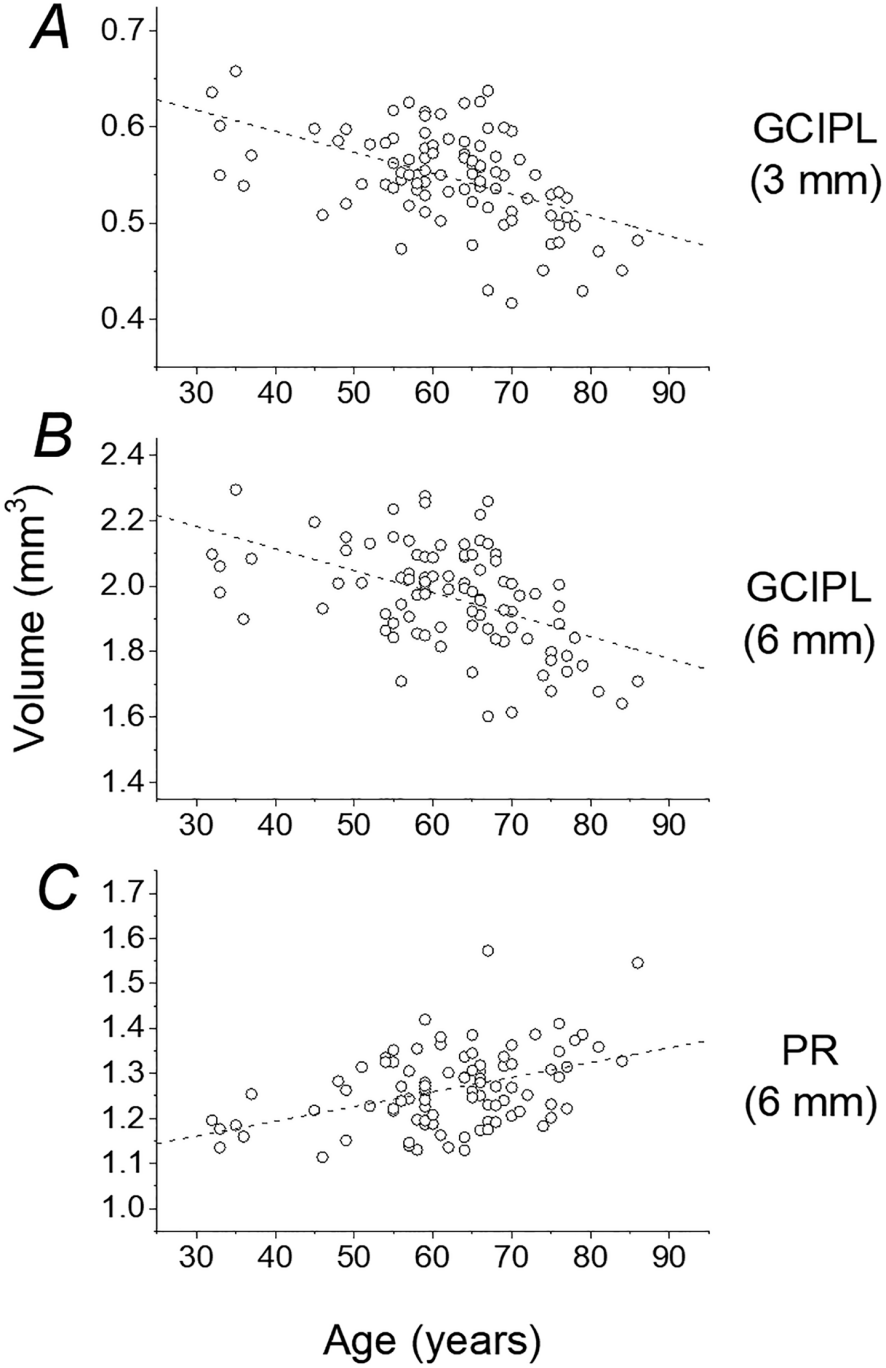
Selected layer volumes plotted as function of age. Points average both twins from each pair. *Dashed lines* show linear fits. (**A**) GCIPL volume in 3-mm diameter central circle. Linear fit declines by 0.022 mm^3^ per decade. (**B**) GCIPL volume in 6-mm diameter circle. Linear fit declines by 0.067 mm^3^ per decade. (**C**) PR volume in 6-mm diameter circle. Linear fit increases by 0.033 mm^3^ per decade.

Significant positive correlations with age were observed for PR (for 3- and 6-mm circles), RPE (3 and 6 mm circles), and OPL (6-mm circle). The age correlations were moderately strong (magnitude >0.4) for GCIPL (for 3 and 6 mm circles) and for PR (6-mm circle). These parameters are plotted against age in Figure 1. Using a simple linear fit, GCIPL volume declined by 0.022 mm^3^ (3-mm circle) and 0.067 mm^3^ (6 mm) per decade. PR volume (6-mm circle) increased by 0.033 mm^3^ per decade.

### Intereye Correlations

The final column of Table 1 gives the intereye correlation coefficient for each parameter. All intereye correlations were highly significant (*P* < 1x10^−8^). Correlations were highest (>0.8) for TRV and GCIPL (both for the 6-mm circle); all segmented layer volumes showed a higher correlation for the 6-mm circle compared with the 3-mm circle.

### Correlations in MZ and DZ Twins and Estimates of Heritability

**Figure 2.**
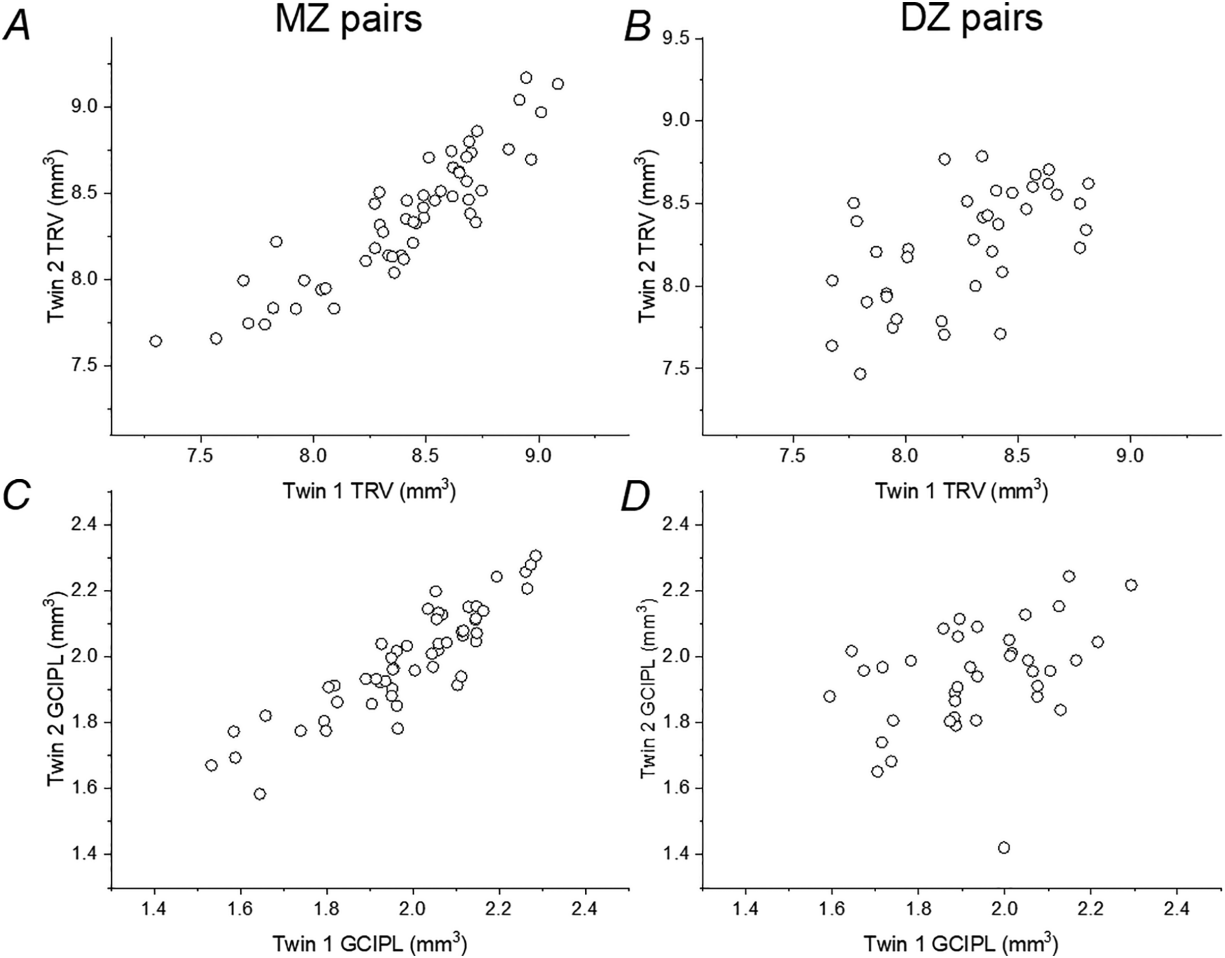
Selected layer volumes plotted for twin pairs (twin 2 is plotted against twin 1). Left-hand panels are for MZ pairs; right-hand panels show DZ pairs. (**A** and **B**) Points plot TRV for the 6-mm diameter circle. (**C** and **D**) GCIPL volume for the 6-mm diameter circle.

**Figure 3.**
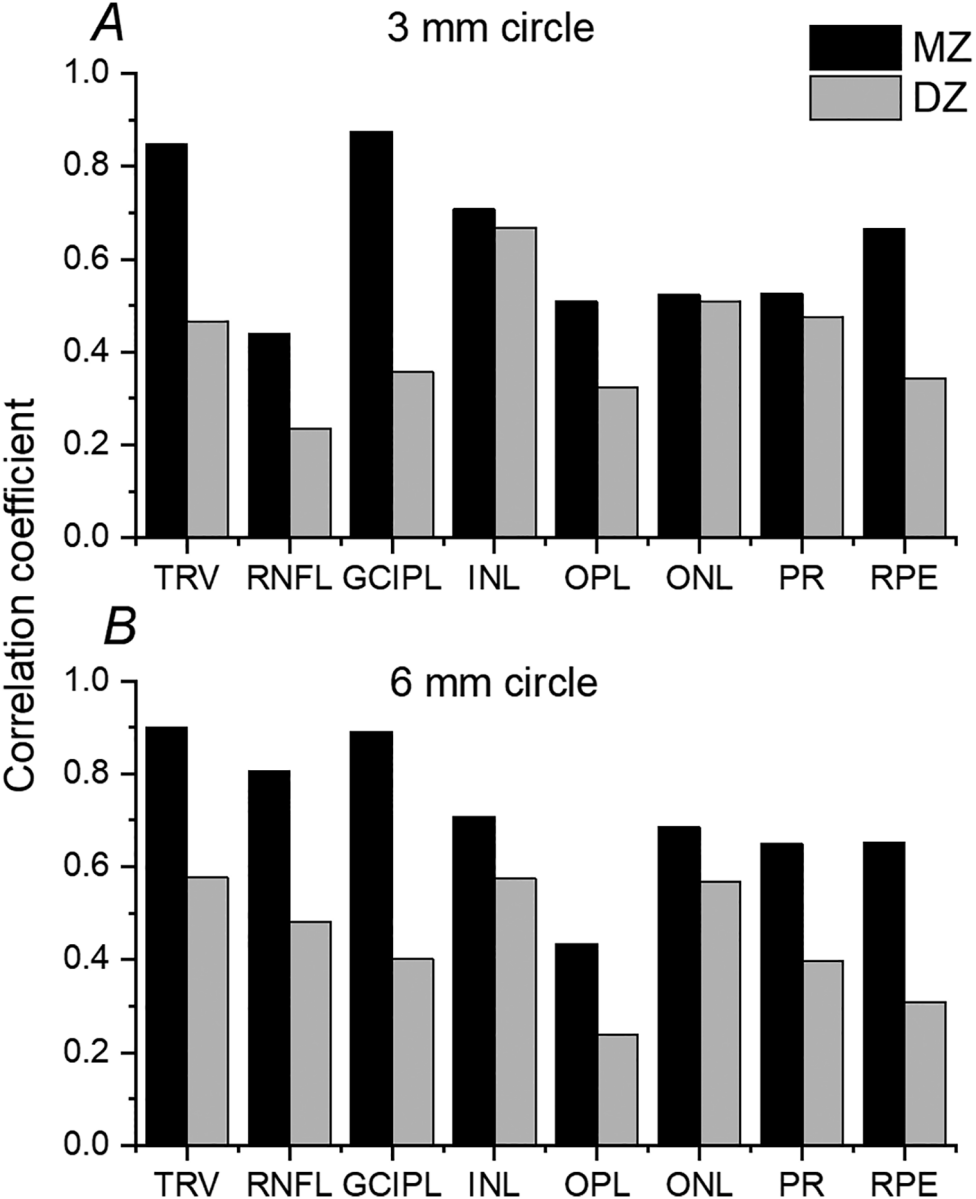
(**A** and **B**) Coefficients for intrapair correlation for MZ and DZ pairs for segmented layer volumes. For RPE, Spearman coefficients are plotted as these volumes deviated from a normal distribution; Pearson coefficients are plotted for all other layers.

Table 2 gives coefficients of intrapair correlation for MZ and DZ twins. The majority were statistically significant, and all were stronger in MZ than DZ twins, consistent with significant heritability. Age-adjusted heritability estimates are also given in Table 2. The majority of parameters appeared to fit best with the AE model. TRV showed high heritability for both the 3- and 6-mm circles (point estimates of 83.0 and 87.5%, respectively). Of the segmented layer volumes, heritability appeared highest for GCIPL (point estimates of 83.7 and 85.8% for the 3- and 6-mm circles, respectively), and lowest for RPE volumes (confidence intervals overlapping zero). Figure 2 plots TRV and GCIPL volumes (for the 6-mm circles) for twin pairs, illustrating the tighter correlation observed in MZ pairs for these parameters. Figure 3 plots MZ and DZ correlation coefficients for all parameters.

**Table 2.** MZ and DZ Coefficients of Intrapair Correlation for Segmented Layer Volumes and Age-Adjusted Estimates of Heritability

		Coefficients of Intrapair Correlations				
		MZ Pairs		DZ Pairs		
Circle						Heritability (%)Point
Diameter (mm)	Parameter	Coefficient	*P* Value	Coefficient	*P* Value	Estimate (95% CI)
3	TRV	0.848*	6.16 × 10^−16^	0.466*	0.003	83.0 (73.9–88.7)
	RNFL	0.440*	8.61 × 10^−4^	0.234	0.158	42.9 (20.5–60.6)
	GCIPL	0.875*	4.84 × 10^−18^	0.357*	0.027	83.7 (74.0–89.6)
	INL	0.707*	2.31 × 10^−9^	0.668*	4.53 × 10^−6^	70.0 (56.2–80.0)
	OPL	0.508*	8.80 × 10^−5^	0.325*	0.047	50.2 (28.9–66.2)
	ONL	0.522*	5.20 × 10^−5^	0.508*	0.001	55.6 (37.2–69.3)
	PR	0.525*	4.65 × 10^−5^	0.474*	0.003	42.9 (22.9–59.1)
	RPE	0.206 (0.664*)	0.136 (4.36 × 10^−8^)	0.104 (0.344*)	0.533 (0.034)	15.4† (<0.1–49.0)
6	TRV	0.899*	2.85 × 10^−20^	0.576*	1.53 × 10^−4^	87.5 (80.7–91.8)
	RNFL	0.806*	2.04 × 10^−13^	0.481*	0.002	71.3 (57.4–80.8)
	GCIPL	0.890*	2.20 × 10^−19^	0.401*	0.013	85.8 (77.4–91.0)
	INL	0.708*	2.18 × 10^−9^	0.574*	1.66 × 10^−4^	71.4† (58.2–80.5)
	OPL	0.434*	0.001	0.238*	0.150	38.3 (15.5–56.9)
	ONL	0.686*	1.02 × 10^−8^	0.568*	2.02 × 10^−4^	69.0† (54.7–78.9)
	PR	0.649*	1.08 × 10^−7^	0.397*	0.014	55.4 (35.4–70.1)
	RPE	0.213 (0.651*)	0.122 (1.01 × 10^−7^)	0.165 (0.307)	0.323 (0.061)	16.3 (<0.1–37.0)

All are Pearson correlation coefficients; for RPE parameters, Spearman coefficients are also given in parentheses as these parameters deviated from a normal distribution. *Denotes significance (*P* < 0.05). Heritability estimates are from the AE model, which provided the best fit for most parameters. †Denotes the following parameters, which showed a marginally better fit with other models: for INL at 6 mm, ACE model generated a heritability estimate of 12.3% (<0.1%–58.0%); for ONL at 6 mm, ACE model generated an estimate of 22.6% (<0.1%–73.6%); for RPE at 3 mm, E appeared the best fitting model.

## Discussion

This study analyzed segmented retinal layer volumes from spectral domain macular OCT scans obtained from 184 twin participants. Means (and SD) were derived for each layer for circular regions of 3 and 6 mm in diameter around the foveal center. TRV, and volumes of inner retinal layers (nerve fiber layer, GCIPL, INL) decreased with age; increasing volume with age was observed for PR, RPE, and OPLs. Intereye correlations were all significant and were highest for TRV and GCIPL volume. Intrapair correlation was greater in all cases for MZ pairs than DZ pairs, and heritability estimates were highest (point estimates >80%) for TRV and GCIPL volume, and lowest for RPE volume.

Reduction in retinal thickness (and nerve fiber layer thinning) with increasing age is well established.^13^ Recently, analysis from the UK Biobank study, confirmed a reduction in macular thickness with increasing age (in fields other than the central 1-mm subfield) from the scans of over 32,000 participants.^14^ The finding in the present study of increasing volume with age in some outer retinal layers (OPL, PR, and RPE) is interesting. The UK Biobank study revealed RPE thinning with age (among those aged older than 45 years),^15^ and it is possible that differences in segmentation methods or population demographics might explain why increase in volume with age was apparent in the present study. The age range for the UK Biobank study was 40 to 69 years, whereas the present study had both younger and older individuals. A positive correlation between foveal RPE thickness and age has been reported by other authors,^16^^,^^17^ and a further study reported, consistent with our findings, a thickening of RPE with age in pericentral and peripheral macular rings.^18^

Increase in PR outer segment volume has been shown in a study of 68 normal eyes,^19^ agreeing with the results of the present study. OPL thickening with age has also been reported in a study of 297 healthy eyes.^20^ Interestingly, the same study reported PR layer thinning with age. It is possible that methodologic differences explain the disagreement with the present study—that study used the Heidelberg Spectralis OCT device (Heidelberg Engineering, Heidelberg, Germany); thickness measurements vary between devices,^21^ and segmentation may also differ.^22^ Increasing OPL layer thickness with age, as found in the present study, was also recently reported in an OCT study of macaque eyes (Renner L, et al. *IOVS* 2019;60:ARVO E-Abstract 202), and OPL remodeling in aged vervet monkeys has been reported from a histological study (Garneau J, et al. *IOVS* 2019;60:ARVO E-Abstract 3102).

As the RPE ages, function, including phagocytosis of PR outer segments, may decline, which might explain an increase in PR layer volume with age. The OPL volume increase might represent remodeling or might also be a consequence of mechanical factors, including expansion as neighboring cellular layers might reduce in volume with age. It is possible that an increase in extracellular space owing to reduction in cellular volume might be contributory.

Our study demonstrated significant heritability of the majority of segmented layer volumes, with point estimates suggesting that 87.5% and 85.8% of the variance in TRV and in GCIPL volume, respectively, could be explained by genetic factors (for the 6-mm diameter central area). Outer retinal layer volumes (especially RPE) appeared to show lower heritability. This could represent a greater influence of, or vulnerability to, environmental factors. It could also relate to greater accuracy in quantification of the inner layers. If outer layer segmentation is less reliable, and more prone to measurement error, then this will manifest as a unique environmental factor, and act to reduce the estimated heritability. Intereye correlation (which can, with limitations, act as a surrogate for repeatability given that both eyes of a healthy individual are highly correlated) was lower for the outer retinal layers, consistent with this notion.

Limitations of the present study include its cross-sectional nature, which make conclusions regarding effects of age not definitive; a longitudinal study with sufficient numbers would be needed to accurately assess change with age. The conclusions are dependent on the accuracy of the segmentation algorithm, and it is possible that different methods might yield differing findings. Segmentation errors are more frequent in the setting of disorders leading to disruption of retinal layer boundaries; as the overwhelming majority of participants had no retinal pathology, the proportion of segmentation errors is likely to be low. Repeatability of intraretinal layer thicknesses has been shown in a previous study to be high with the segmentation software used (although that study did not evaluate RPE thickness measurements).^23^ However, there has been no formal repeatability study for use of this segmentation software with the Topcon OCT device used in the present study. Nevertheless, the finding of strong interocular correlation for most layers in the present study suggests that, for these layers, the segmentation is likely to have a significant level of reproducibility.

No adjustments were made for axial length or refraction. Differing axial lengths between participants would mean that the actual sizes of the nominal “3-mm” or “6-mm” circular areas would differ. Thus the findings of the present study are better taken as exploring heritability and correlations for layer volumes corresponding to the equivalent angle in degrees rather than lateral extent in millimeters.

A larger sample size would add power and help narrow the confidence intervals of the heritability estimates and establish whether those correlations with age that did not achieve significance in the present study might still represent true correlations. For example, ONL volume for the 6-mm circle appeared to correlate weakly and negatively with age, but this did not achieve significance (correlation coefficient –0.16, *P* = 0.12). If this were a true correlation of this magnitude, then a sample size of 150 independent observations (unrelated individuals) would be estimated to be required to achieve significance (two-tailed *P* < 0.05). Finally, the TwinsUK cohort was largely women and of European descent, thus potentially limiting generalizability to other demographics.

## Conclusions

In summary, our cross-sectional study found that TRV and a number of inner retinal layer volumes were smaller in older participants, whereas some outer volumes appeared to increase with age. Intrapair correlations were greater in MZ than DZ pairs for all layers. Estimated heritability was highest (>80%) for TRV and for GCIPL volumes.
